# Transient central precocious puberty: a new entity among the spectrum of precocious puberty?

**DOI:** 10.1186/s13052-021-01163-9

**Published:** 2021-10-23

**Authors:** Valentina Assirelli, Federico Baronio, Rita Ortolano, Giulio Maltoni, Stefano Zucchini, Valeria Di Natale, Alessandra Cassio

**Affiliations:** 1 Program of Endocrine-Metabolic Diseases, Unit of Pediatrics, University of Bologna, IRCCS- University Hospital of Bologna, Via Massarenti 11, Bologna, Italy; 2 Specialty School of Paediatrics - Alma Mater Studiorum, Università di Bologna, Bologna, Italy

**Keywords:** Central precocious puberty, Endocrine disruptors, Thelarche, Transient precocious puberty, Nutritional factors, Herbicides and pesticides

## Abstract

**Objective:**

Recently, we observed some cases of Precocious Puberty (PP) with a partial central activation of hypothalamic-pituitary-gonadal (HPG) axis that tended to normalized in 6–12 months. To evaluate the frequency of this form within the spectrum of forms of PP, we retrospectively assessed the clinical, hormonal and ultrasound characteristics of patients attending to our Center for signs of PP, between 2007 and 2017. To hypothesize some causes of this “pubertal poussée” a questionnaire about environmental data was provided to patients.

**Methods:**

96 girls were recruited for the study and divided into three Groups. Group 1: 56 subjects with Central PP (CPP) requiring treatment with GnRH analogue; Group 2: 22 subjects with transient activation of pubertal axis, that tended to normalize, “Transient CPP”(T-CPP); Group 3: 18 subjects with Isolated Thelarche (IT).

**Results:**

Mean age at diagnosis was 6.8 ± 1.0 years in Group 1, 5.9 ± 1.3 years in Group 2 and 5.6 ± 1.5 years in Group 3. A significant increase of diagnosis of T-CPP was observed over the study period. Significantly higher use of some homeopathic medicines and potential exposure to pesticides was reported in Group 2 vs Group 1**.**

**Conclusions:**

To our knowledge, we first reported a form defined as T-CPP, characterized by partial activation in the HPG axis normalizing over time. An increased use of homeopathic medicines and exposure to environmental pollutants in these patients was evidenced.

## Introduction

Precocious puberty (PP) in girls is defined as the onset of thelarche before 8 years of age [1].

The onset of puberty depends on many factors, such as family history, low birth weight, obesity in infancy and early childhood, international adoption (with a risk of 10–20 times higher), and also exposure to endocrine-disrupting chemicals (EDCs) has been implicated. However, the influence of these factors in the changes of pubertal onset is still controversial [2, 3].

Within idiopathic forms, Central Precocious Puberty (CPP) does not represent a single entity, but rather a spectrum of forms ranging from Isolated Thelarche (IT) to rapidly progressive CPP [4]. Stanhope et al. described for the first time a variant of PP characterized by thelarche and acceleration of growth rate. This form was called Thelarche Variant (TV), as it did not develop in CPP and had no response to GnRH analogue [5–7]. Among all these forms of non progressive or slowly progressive PP, characterized by stabilization or slowly progression of pubertal signs not requiring treatment [8], the degree of activation of Hypothalamic-Pituitary-Gonadal (HPG) axis and his evolutionary trend have never been clarified. Recently, in our center, we observed some cases of PP characterized by a partial activation of HPG axis that not only didn’t progress, but tended to regress and normalized. In order to evaluate the frequency of this form within the spectrum of forms of PP, we retrospectively assessed the clinical, hormonal and ultrasound characteristics of consecutive patients attending for signs of PP to our Center between 2007 and 2017.

We also used a questionnaire to evaluate the possible role of socio-economic, anamnestic, nutritional and environmental factors on the onset of these forms.

## Patients and methods

### Patients and study design

We evaluated retrospectively 96 girls referred to our Center for suspected PP between 1st January 2007 and 31th December 2017.

Inclusion criteria were: age at onset of pubertal signs between 3 and 8 years, availability of blood sampling for estradiol, basal and after stimulation gonadotropins, availability of Bone Age (BA) and Pelvic Ultrasound, negative brain MRI in treated girls and at least a one-year-follow up after diagnosis.

The exclusion criteria were: isolated pubarche and other genetic or neurological syndromes including CPP.

According to the GnRH test response, the ultrasound data and BA/Chronological Age (CA) ratio at diagnosis, girls were divided into three Groups:

- Group 1: 56 subjects with idiopathic progressive CPP who required treatment with GnRH analogue (Triptorelin Depot 3.75 mg every 4 weeks by intramuscular injection), according to Consensus Criteria [9] [10]
- Group 2: 22 patients with a form of CPP characterized by an intermediate response of LH at the GnRH test (arbitrarily defined as a LH peak between 3 and 5 IU/mL considering that a LH peak > 5 IU/mL is the usual marker of a complete HPG axis activation [11]), advancement of BA no more than 1 year compared to CA, longitudinal uterine diameter > 36 mm and/or uterine volume > 3.5 ml. These parameters have moreover normalized until 6–12 months of follow-up. We defined this form as Transient CPP (T-CPP).
- Group 3: 18 patients with IT without any other sign of central activation of the HPG axis.

### Methods

For all patients we retrospectively investigated from medical records: familiar history, socio-economic condition, pregnancy and delivery, age at mother’s menarche, urban or rural residence. Subjects were asked to reply to a questionnaire, sent by post, investigating the use of assisted reproduction techniques, use of drugs during pregnancy, breastfeeding, exposure to smoking, use of herbal supplements or homeopathic drugs, eating habits, use of soy-based products, distance < 1000 m from an intensive farming or an industry capable of exposing to environmental pollutants or use of cosmetic products containing placenta extracts.

**Fig. 1:**
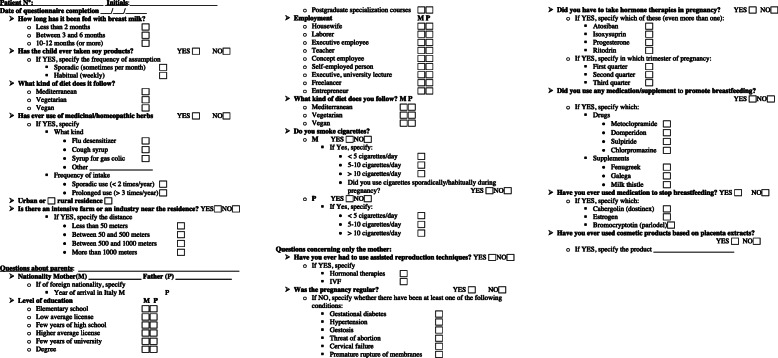
Questionnaire administered to families, concerning socio-economic, anamnestic, nutritional and environmental aspects

The questionnaire details are shown in Fig. 1.

Overall 50/96 subjects (about 50%) completed the questionnaire, 34/56 (60.7%) in Group 1 and 16/22 (72.7%) in Group 2. In Group 3, only 2 patients have accepted to complete the questionnaire, so these data were not included into the study.

Pubertal development was assessed according to Tanner and Whitehouse’s criteria [12]. The height was measured using Harpenden stadiometer and expressed as Standard Deviation Score (SDS). BA was evaluated using the atlas of Greulich and Pyle [13]. The BMI was calculated according to the formula: weight (kg) / height (m2), and the BMI SDS was calculated according to age and sex. The socio-economic condition was calculated according to the Socio-Economic Status of Hollingshead, taking into account the level of education and the profession of both parents (Hollingshead: Four factor index of social status. In: New Haven Department of sociology, Unpublished).

Blood samples were collected in fasting conditions between 07.00 and 09.00 in the morning. The levels of gonadotropins (LH and FSH) and 17-β estradiol were analyzed by an immunochemiluminometric method (ICMA, Axsym Abbott) with a sensitivity threshold of 0.1 IU/mL for gonadotropins. The GnRH test was performed at diagnosis in all patients and then repeated at 6–12 months in Groups 2 and 3. In the patients of Group 1, after starting the therapy, only baseline gonadotropin levels were checked.

The conventional GnRH stimulus test was performed by intravenous administration of 50–100 μg of drug, with samples taken at 0′, 30′, 60′ [9, 14].

The levels of TSH and FT4 were evaluated by means of a chemiluminescent assay (Bayer, Fenwald, Germany) (normal range: TSH 0.5–4.5 mU/L, FT4 9–17 pg/mL). Pelvic ultrasound was performed trans-abdominally by a group of radiologists expert in pediatric evaluations [15]. It was used a Convex ecotomograph with B-mode ultrasound signal and a wavelength variable between 3.5 and 5.0 MHz (Philips). By evaluating the subject in a supine position and with a distended bladder, were made uterine measurements (longitudinal diameter (LD), transverse diameter (TD) and antero-posterior diameter (APD)), ovarian measurements and was the evaluated the visibility of the endometrial rhyme. The uterine volumes were obtained through the ellipsoid formula (DL x DT x DAP × 0.52) / 1000.

For statistical analysis, categoric variables were presented as frequencies or percentages; continuous variables with normal distribution were presented as mean values ± SDS. The Kruskal Wallis test with independent samples was used to evaluate the normal distribution of the parameters. The Chi-Squared test was used to evaluate whether or not to reject the null hypothesis, using a 95% confidence interval. The Mann Whitney test was used to verify the differences between independent groups. A value of *p* < 0.05 was considered significant in all cases. Statistical analyzes have been performed using STAT program for Windows.

Our study was approved by our ethics committee of the hospital (197/2016/O/Oss) and written informed parental consent was obtained before the start of the study.

## Results

**Fig. 2:**
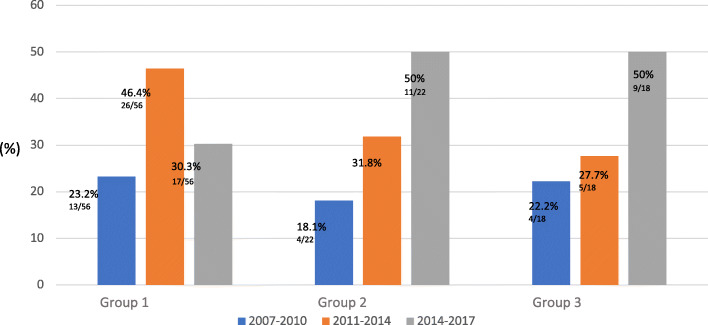
Distribution of the diagnosis (%) of the different forms of PP in our Center between 2007 and 2017

The distribution of the different forms of PP diagnosed in our Center from January 2007 to December 2017 is shown in Fig. 2.

We observed a significant increase in Group 2 and Group 3 diagnosis over the study period (*P* < 0.05).

Table 1 shows some auxological and anamnestic features at the first evaluation in the groups of girls examined.

**Table 1:** Auxological and anamnestic data in the 3 groups of girls at the first evaluation

	Group 1 (n° 56)	Group 2 (n° 22)	Group3 (n°18)
CA at onset of pubertal signs	7.00 ± 1.00*	6.35 ± 1.20°	4.85 ± 1.90
CA diagnosis	7.58 ± 0.73§	6.75 ± 1.06	6.25 ± 0.35
BA diagnosis	9.55 ± 1.00§	7.20 ± 1.70	7.94 ± 1.04
Height at diagnosis (SDS)	1.19 ± 0.82§	0.50 ± 1.20	0.80 ± 0.76
BMI at diagnosis	0.56 ± 0.90	0.29 ± 0.90	0.98 ± 0.69**

*p < 0.05 vs Group 3; ° *p* < 0.05 vs Group 3; §*p* < 0.05 vs Group 2 and 3; **p < 0.05 vs Group 2

*CA* Chronological Age, *BA* Bone Age

Caucasian race was reported in 51/56 (91%) girls in Group 1, 20/22 (90%) in Group 2 and 16/18 (88.8%) in Group 3. Seven girls were adopted from foreign countries, 6/56 (10%) in Group 1 and 1/22 (4.8%) in Group 2. They were adopted at an average CA of 2.42 ± 3.10 years (3.4 ± 1.2 years before the onset of pubertal signs). In Group 2 we also found 2/22 (9%) girls born from mothers emigrated from Russia and South America about 2 years before conception. We did not find comparable cases in Groups 1 and 3. There were no statistical differences regarding familiarity for PP. Group 3 shows a significantly lower neonatal weight than Group 2 (2870 ± 634 g vs 3270 ± 546 g) (*p* < 0.05), without differences from Group 1.

**Fig. 3:**
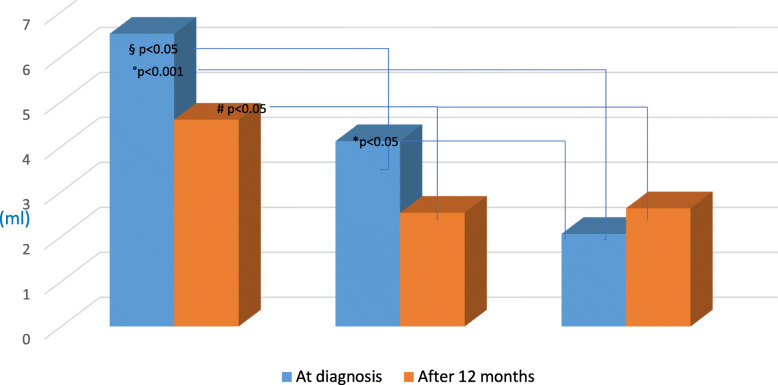
Mean Uterine Volume at Diagnosis and at 12 months of Follow-up in the 3 Groups examined. §*p* < 0.05 vs Group 2 at diagnosis, ° *p* < 0.001 vs Group 3 at diagnosis, * *p* < 0.05 vs Group 3 at diagnosis, # *p* < 0.05 vs Group 2 and 3 at 12 months of follow up

Table 2 and Fig. 3 showed laboratoristic and ultrasound data at diagnosis and at 6–12 months of follow-up in the groups of girls examined.

**Table 2:** Laboratoristic and ultrasound data at diagnosis and at 6–12 months of follow up in the Groups of girls

	At diagnosis				At 6–12 months of follow up			
	Baseline LH (U/mL)	Peak LH (U/mL)	LH/FSH peak ratio	ULD (mm)	Baseline LH (U/ml)	Peak LH (U/ml)	LH/FSH peak ratio	ULD (mm)
Group 1	1,4 ± 2.3*	11.7 ± 7.8*	1.1 ± 0.7*	42.5 ± 6.3*	0.6 ± 0.8			40.2 ± 6.3#
Group 2	0.1 ± 0.1	4.6 ± 1.6§	0.3 ± 0.1	36.5 ± 4.9§	0.3 ± 0.8	2.3 ± 1.3°	0.3 ± 0.1	35.1 ± 4.2
Group 3	0.1 ± 0.0	2.2 ± 1.1	0.2 ± 0.0	30.7 ± 4.7	0.1 ± 0.0	1.8 ± 0.1	0.1 ± 0.0	32.2 ± 2.7

*ULD* Uterine Longitudinal Diameter

*p < 0.001 vs Group 2 and 3; § *p* < 0.05 vs Group 3; #*p* < 0.05 vs Group 2 and 3; °*p* < 0.05 vs Group 2 at diagnosis

Hormonal and ultrasound data at diagnosis are consistent with the study design. At 6–12 months, LH peak levels of Group 2 were similar to Group 3 and significantly lower compared with LH peak at diagnosis.

In Group 1, patients started Triptorelin therapy at an average CA of 7.58 ± 0.73 years. The therapy was suspended at mean CA of 10.1 ± 0.60 years (mean BA of 11.5 ± 0.7 years). The age at menarche was 11.4 ± 0.9 years (age of maternal menarche 11.08 ± 1.28).

Group 2 and 3 patients were followed for 24 ± 4.1 months and 24 ± 3.2 months respectively. The age at menarche was 12 ± 0.7 years in Group 2 (age of maternal menarche 12.45 ± 1.25). The age at menarche for Group 2 was extrapolated from the questionnaire responses and was available for 5/22 girls.

Estradiol levels were undetectable in 20/56 (35%) in Group 1 and in all patients of other Groups. TSH values above 4.5 mU/L was reported at diagnosis in 4/22 patients of Group 2 (18%) and 2/18 patients of Group 3 (11%), and in no subject from Group 1. No subjects showed a TSH level > 10 mU/L. FT4 levels were always in the normal range, and thyroid antibodies were always negative.

About ultrasound parameters, after 12 months there was no longer any statistically significant difference on uterine measurement between Groups 2 and 3 (Table 2, Fig. 3).

In Table 3, we showed socio-economic, anamnestic, nutritional and environmental data in the two groups of girls who answered the questionnaire.

**Table 3:** Socio-economic, anamnestic, nutritional and environmental data derived from the questionnaire in the responder girls

	Group 1 (34/56 responders)	Group 2 (16/22 responders)
**Questionnaire data**	34/56	16/22
**Hollingshead SSE**	1,2 ± 0.4	1.3 ± 0.7
**Rural Residency**	14/34 (41.2%)	5/16 (31.2%)
**Urban Residency**	20/34 (58.8%)	11/16 (68.8%)
**Smoking Mother**	6/34 (17.6%)	3/16 (18.7%)
**Smoking Father**	6/34 (17.6%)	2/16 (12.5%)
**Mediterranean Diet**	32/34 (94.1%)	15/16 (93.7%)
**Vegetarian Diet**	2/34 (5.9%)	1/16 (6.3%)
**Soy-products**	3/34 (8.8%)	0/16 (0%)
**AFT**	1/34 (2.9%)	0/16 (0%)
**Breastfeeding**	19/34 (55.8%)	8/16 (50%)
**Integrator during breastfeeding**	0/34 (0%)	0/16 (0%)
**Homeopathic medicine**	16/34 (47%)	8/16 (50%)
**Homeopathic cough syrup**	5/34 (14.7%)	4/16 (25%)*
**Homeopathic solution for gas colic**	0/34 (0%)	1/16 (6.25%)*
**Herbicides and Pesticides exposition**	4/34 (11.7%)	3/16 (18.7%)§
**Cosmetics containing placenta extract**	2/34 (5.9%)	2/16 (12.5%)*

*AFT* assisted fertilization technique

*p < 0.05 vs Group 1. §*p* < 0.001 vs Group 1

There were no differences between the groups for the following data: residency, use of techniques for assisted fertilization (hormonal therapies or in vitro fertilization), breastfeeding, use of supplements to promote breastfeeding, exposure to smoking, food containing soya, drugs during pregnancy and socio-economic condition.

As for homeopathic drugs, in Group 2 was reported a significantly higher use of a homeopathic cough syrup and a natural solution for the gas colic than in Group 1. These medicines contain different types of herbal extracts, in particular *Foeniculum Vulgare* in the second one, whose effects on pubertal axis are not still clarified, but a certain estrogen-stimulating activity has been reported [16].

In Group 2 was reported a significantly higher potential exposure to herbicides and pesticides than in Group 1, expressed as a distance < 1000 m from an intensive farming or industry.

## Discussion

It is well known that idiopathic CPP does not represent a single entity, but rather a spectrum of forms ranging from IT to rapidly progressive CPP [4]. As above mentioned, Stanhope et al. [6] described for the first time a variant of PP characterized by thelarche with acceleration of growth rate. This form was called TV, as it did not develop in CPP and had no response to GnRH analogue treatment [5] [6] [7]. Among all these forms of non progressive or slowly progressive PP, characterized by stabilization or slow progression of pubertal signs not requiring treatment [8], the degree of activation of HPG axis and his evolutionary trend have never been clarified. Alongside these forms, our results seems to reveal a form of CPP characterized by partial activation of the HPG axis that not only did not progress, but tended to regress and normalized during the follow-up.

To the best of our knowledge, we reported for the first time a form of CPP that we have defined as T-CPP. Although the similar to TV, previously described in literature [7], T-CPP is characterized by a partial central activation of pubertal axis, as demonstrated by hormonal and ultrasound features, able to normalize over time without any pharmacological treatment. The onset of pubertal signs and their subsequent normalization differentiates this form from the behaviour of non progressive or slowly progressive CPP [3].

Furthermore, there are still very controversial data in literature on the possible factors involved in the etiology of the various forms of pubertal advancement, in particular with regard to environmental factors [17–19].

The pathogenesis of this variant appears unclear and may not be univocal. The role of EDCs on the development of PP is still under discussion, because pubertal timing, and subsequently the onset of puberty, can be strongly affected by [17–20].

The results of questionnaire were obtained by interviewing a small percentage of patients; therefore, these data can hardly be considered representative. Even with this limit, the results suggest a partial influence of some environmental factors. In fact, subjects in Group 2 reported an increased use of homeopathic medicines and exposure to environmental pollutants. The method of questionnaire administration does not allow us to establish how long before the appearance of pubertal signs the exposure to homeopathic drugs took place, so without this information we can only speculate that these substances may have a transient estrogen stimulating action.

We found a significantly lower neonatal weight in Group 3 compared to Group 2. This result could be of non-univocal interpretation. In literature, it is described how intrauterine growth retardation may be a potential risk factor for PP or premature pubarche, but this is not clearly described for premature thelarche.

Surprisingly, we found a higher frequency of hyper-thyrotropinemia than in general population (2%) [21]. Several reports about forms of early pseudopuberty in subjects with primitive hypothyroidism have been already published [21]; this phenomenon was partially explained by the structural homology between TSH and gonadotropins, in particular FSH, with consequent activation of ovarian estrogen receptors. However, in literature is reported that the physiologic baseline event in premature thelarche is the increase in FSH level. Inhibin B secreted from granulosa cells is thought to be responsible for this increase [22].

Regarding adoption, Soriano et al. revealed an association between adoption and a precocious central activation of the HPG axis [23]. In our case series we found only 7 patients adopted, 6 of which, however, in group 1. In any case, considering the small number of subjects adopted on the total sample, it becomes difficult to confirm or not Soriano’s conclusions. As for the incidence of girls born in Italy from mothers migrated from other countries, the association with an early pubertal development is currently being evaluated in literature, as a consequence of stressful social factor, but also improvement in quality of life and precedent exposure to environmental interferents [24].

The results of our study showed changes of frequency of the different forms of CPP over years. On one hand, the diagnosis of rapidly progressive forms decreased over time, probably due to a better clinical selection of patients, on the other, a significant increase in non-progressive forms was observed. This appears in partial disagreement with a recent Danish study deriving from a population registry, which indicates an increase not only in benign pubertal variants (pubarche and IT) but also in CPP. Moreover, the two studies are difficult to compare due to different characteristics and study design [25].

The strength of our study is that subjects were followed by a single center with a homogeneous diagnostic approach.

There are many limits in our study: the retrospective study design, the small sample size, the poor compliance to the questionnaire, the retrospective ultrasound evaluation, the absence of a control group and the indirect evaluation by questionnaire of possible etiological role of EDCs. In particular, the risk of an intra-interoperator variability can affect the reliability of the results of retrospective ultrasound evaluation. However we must underline that all the operators involved in this practice were pediatric radiologists with considerable experience in this topic.

In the future, multicenter prospective studies, supported also by laboratory measurement of EDCs, will be necessary to confirm the frequency, the characteristics and the evolution of T-CPP and to evaluate the possible role of environmental causes on “pubertal poussée”.

## Conclusions

The results of our study showed over time a decrease in the diagnosis of rapidly progressive forms, associated instead with a significant increase in non-progressive forms. To the best of our knowledge, we have reported for the first time a form defined as T-CPP, characterized by a partial activation of the HPG axis which normalizes over time. The pathogenesis of this variant appears unclear and may not be univocal. The results of the questionnaire, even if not very representative, suggest a partial influence of some environmental factors, such as homeopathic medicines and environmental pollutants.

## Data Availability

Data are available upon reasonable request.

## Abbreviations

PP: Precocious puberty
HPG: Hypothalamic-pituitary-gonadal
CPP: Central precocious puberty
T-CPP: Transient-central precocious puberty
IT: Isolated thelarche
EDCs: Endocrine disrupters
TV: Thelarche variant
BA: Bone age
CA: Chronological age
SDS: Standard deviation score
LD: Longitudinal diameter
ULD: Uterine longitudinal diameter
TD: Transverse diameter
APD: Antero-posterior diameter
AFT: Assisted fertilization technique
